# A Room Temperature H_2_ Sensor Fabricated Using High Performance Pt-Loaded SnO_2_ Nanoparticles

**DOI:** 10.3390/s150614286

**Published:** 2015-06-17

**Authors:** Sheng-Chang Wang, Muhammad Omar Shaikh

**Affiliations:** Department of Mechanical Engineering, Southern Taiwan University of Science and Technology, Tainan 710, Taiwan; E-Mail: omar.offgridsolutions@gmail.com

**Keywords:** gas sensor, thick film, porous microstructure

## Abstract

Highly sensitive H_2_ gas sensors were prepared using pure and Pt-loaded SnO_2_ nanoparticles. Thick film sensors (~35 μm) were fabricated that showed a highly porous interconnected structure made of high density small grained nanoparticles. Using Pt as catalyst improved sensor response and reduced the operating temperature for achieving high sensitivity because of the negative temperature coefficient observed in Pt-loaded SnO_2_. The highest sensor response to 1000 ppm H_2_ was 10,500 at room temperature with a response time of 20 s. The morphology of the SnO_2_ nanoparticles, the surface loading concentration and dispersion of the Pt catalyst and the microstructure of the sensing layer all play a key role in the development of an effective gas sensing device.

## 1. Introduction

Since hydrogen (H_2_) is currently used in many different sectors of technology and is expected to be the fuel of the future, a H_2_-selective sensor material operating at room temperature is of considerable immediate interest. H_2_ is known to be a flammable and explosive gas that is undetectable by the human senses and thus sensors that can detect low concentrations of it in the event of a leak must be fabricated. In general, gas sensors are of great interest because of their ability for real time analysis of gaseous chemicals in a wide range of applications [1,2,3,4,5]. Solid-state gas sensors, especially those that are based on metal oxide semiconductors have been widely utilized owing to their low cost, simple crystal structure, ease of integration, excellent stability, high sensitivity and rapid response. The sensing properties of these sensors are determined by the redox interaction of the gas molecules with the metal oxide surface. Sensors fabricated using nanocrystals of these semiconductor oxide materials have been shown to exhibit a quicker response at lower H_2_ concentrations owing to the large surface area achieved at the nanoscale. In particular, tin dioxide (SnO_2_) is a well-researched gas sensing material which has been studied in various morphologies for detection of a wide range of gases [6,7,8,9,10,11,12]. SnO_2_ in its bulk form is a colorless, diamagnetic, wide bandgap n-type semiconductor that crystallizes in the rutile tetragonal structure of the mineral cassiterite and is the best understood oxide material for the detection of reducing gases. SnO_2_ thin and thick film gas sensors have been made using various techniques like sputtering, chemical vapor deposition, spray pyrolysis and sol-gel methods [13,14,15,16]. Research on the optimal design of oxide nanoparticle (NP)-based gas sensors has shown that the sensitivity and the sensing response can be simultaneously and significantly enhanced when the particle size is close to twice the thickness of the carrier depletion layer. However, it has been observed that massive aggregation occurs either during NP synthesis or during the film casting process which greatly reduces the surface area and gas transporting pathways and thus seriously suppresses the sensing performance. A porous interconnected configuration in sensing layers is considered as the most fundamental strategy to efficiently provide larger active surface areas for gas sensors. However, the precise control and integration of the desired porous structure using bottom-up techniques still remains a challenge. While high sensitivity to selective gases has been achieved at higher operating temperatures, ultrasensitive room temperature operating H_2_ gas sensors is an area still under active research. To date, it has been experimentally proven that the addition of noble metals like gold (Au), palladium (Pd) and platinum (Pt) as surface additives to the semiconducting oxides improves sensor response and recovery time while simultaneously decreasing the operating temperature [17,18,19,20,21]. In particular, Pt has been observed to be an effective catalyst in detection of H_2_ by enhancing the rate of chemical sensitization via the spill-over mechanism [22,23,24,25] which greatly promotes the dissociation of oxygen molecules and increases the ionosorption of the dissociated oxygen species on the surface of the SnO_2_ NPs. At room temperature, this catalytic effect greatly withdraws electrons from the conduction band of the SnO_2_ NPs, widening the electron depletion region and leading to higher surface Schottky barriers. When the Pt-loaded SnO_2_ NPs are exposed to H_2_ gas, the Pt catalyzes the oxidation of H_2_ which causes the electrons trapped by the dissociated oxygen species to be released back into the conduction band of the oxide, resulting in a drastic fall in the electrical resistance and thus an enhanced sensor response. Therefore, it is reasonable to conclude that with the assistance of the catalytic metals, the semiconductor oxide surfaces are strongly activated to drive the gas sensing reactions required at room temperature. The performance of existing commercially available thick film SnO_2_ gas sensors can be further improved by having greater control over the particle size and the microstructure of the sensing layer. Moreover, pure SnO_2_-based gas sensors are known to have a high resistance and are not effective for making devices that can operate at low temperatures. In this study, we have developed a simple thermal decomposition method for the synthesis of nearly monodisperse SnO_2_ and Pt-loaded SnO_2_ nanoparticles. These nanoparticles are mixed with a solvent and binder to form a nanoparticulate ink which is then screen printed as a thick film and after being annealed at suitable temperatures, results in a porous and well connected microstructure. We have compared the sensor response of the pure and Pt-loaded SnO_2_ thick films at operating temperatures of 400 °C down to room temperature. It was observed that the Pt catalyst not only improved sensor response as compared to pure SnO_2_ nanoparticles, but also showed high sensitivity at room temperature.

## 2. Experimental Section

### 2.1. Materials

Tin oxide (SnO, 99%), platinum chloride (PtCl_2_, 98%), oleic acid (OA, C_18_H_34_O_2_, 66%–88%), tri-*n*-octylamine (TOA, (CH_3_ (CH_2_)_7_)_3_N, 98%), ethyl cellulose (48% ethoxy content), α-terpineol (C_10_H_18_O, 99%), hexane (C_6_H_14_, 90%) and ethanol (H_3_CH_2_OH, 99%) were purchased form commercial sources and used without further purification.

### 2.2. Synthesis of Pure SnO_2_ Nanoparticles

In a typical procedure, SnO (1 mmol) and OA (3 mmol) were heated in a three necked flask at 300 °C for 75 min under argon gas with constant magnetic stirring. The resulting mixture was cooled down to room temperature, washed with ethanol and hexane and centrifuged at 5000 rpm to obtain the tin precursor, tin oleate (Sn(OA)_x_). Once the tin precursor is obtained, one can proceed with the synthesis of the SnO_2_ nanoparticles. Tin oleate precursor (1 mmol), TOA (16 mmol) and OA (4 mmol) were added to a three necked flask. The resulting mixture was heated to 340 °C in air and held there for 3 h. Once cooled to room temperature, the mixture was washed with ethanol and hexane in a centrifuge at 5000 rpm/min and vacuum dried to obtain the pure SnO_2_ nanoparticles.

### 2.3. Synthesis of Pt-Loaded SnO_2_ Nanoparticles

In a typical procedure, the synthesized pure SnO_2_ nanoparticles (1 mmol) and OA (10 mmol) were mixed with varying concentrations of PtCl_2_ in a three necked flask and heated in air to 290 °C at a rate of 10 °C/min and held there for 1 h. Three different concentration of PtCl_2_ were used and the reaction parameters are shown in Table 1. The resulting solution was allowed to cool down, washed with ethanol and hexane in a centrifuge at 10,000 rpm/min, dried and sent for further characterization.

**Table 1:** Experimental reaction conditions for pure and Pt-loaded SnO_2_ nanoparticles.

Sample	Atmosphere	SnO_2_ Solution	Surfactant OA (mmol)	PtCl_2_ Powder (mmol)	Temperature/Time (°C/h)	Average Crystal Size (nm)
(a)	Air	1	0	**0**	340/2	**5.5**
(b)	Air	1	10	**0.05**	290/1	**5.1**
(c)	Air	1	10	**0.1**	290/1	**6.3**
(d)	Air	1	10	**0.15**	290/1	**5.7**

### 2.4. Sensor Fabrication and Measurements

In this study, we have fabricated a porous thick film sensor made of an ink containing pure or Pt-loaded SnO_2_ nanoparticles (40 wt. %) and a paste (60 wt. %). The paste consisted of terpineol (solvent) and ethyl cellulose (binder) in a weight ratio of 93:7. The nanoparticulate ink was screen printed on an alumina (Al_2_O_3_) substrate interdigitated with Pt electrodes (0.4 cm × 0.5 cm). The coated substrates were then annealed at 500 °C for an hour for binder removal before being sent for gas sensing measurements. To improve their stability and repeatability, each sensor was aged in air at 100 °C for 10 days before being used for gas sensing. Furthermore each sensor was tested for repeatability (over several cycles) and stability (over a period of 2 months with measurements made every 10 days). The gas sensing properties were studied in a quartz tube that was placed inside a furnace. The operating temperature was varied from room temperature (R.T. = 25 °C) to 400 °C while the humidity was maintained at 60%–65%. The dry synthetic air was introduced into the quartz tube at a flow rate of 2 L/min while small amounts of H_2_ were precisely added to obtain the desired concentration in synthetic air. In our study, the lowest H_2_ concentration that we can precisely control is 1000 ppm and hence we measure our sensor sensitivity and response at this concentration. Voltage was applied to the sensor (10 V across the Pt electrodes) and the resistance in air (R_a_) and in the presence of H_2_ gas (R_g_) was measured using an Agilent multimeter. The sensor sensitivity (S) is defined as the ratio of the resistance in air to H_2_ (R_a_/R_g_) and is the opposite when calculated for oxidizing gases. The response time (R) is defined as the time needed to obtain a 90% drop in resistance from the equilibrium value after injection and removal of H_2_. The sensors made of pure SnO_2_ and loaded with 0.05, 1.0 and 1.5 mmol PtCl_2_ were designated as Sensor A, B, C and D, respectively.

### 2.5. Structural and Morphological Characterization

X-Ray diffraction (XRD) measurements were made on a D2 Phaser diffractometer (Bruker, Germany) with CuK_α_ radiation (λ = 1.54060 Å) at a scan rate of 0.025 s^−1^ to analyze the phase structure of the nanocrystals and the sensing film. High resolution transmission electron microscopy (HRTEM) was performed on a field emission gun transmission electron microscope (Tecnai G2 G20 FEG-TEM, Philips, The Netherlands) operating at 200 kV. The dried powdered samples were dispersed in hexane to an appropriate concentration and then drop coated on ultrathin carbon coated copper grids and dried in the oven at 80 °C for an hour before being sent for TEM analysis. Surface and cross-sectional morphology of pure and Pt-loaded SnO_2_ thick films were observed using a scanning electron microscope (SEM, JSM-6701F, Jeol, Japan).

## 3. Results and Discussion

### 3.1. Pure SnO_2_ Nanoparticle Based Sensor 

**Figure 1:**
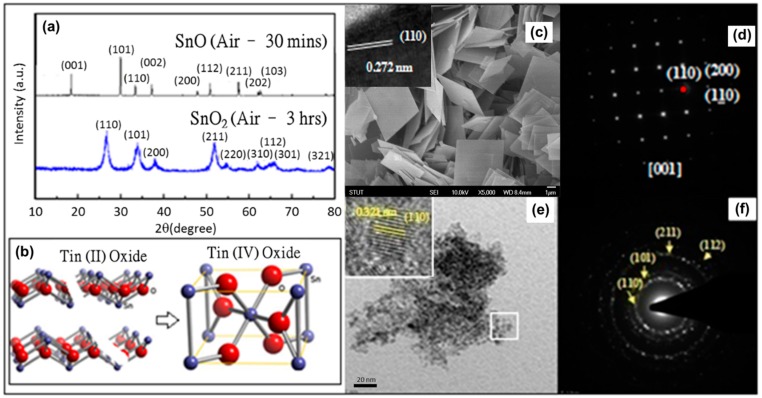
(**a**) XRD of nanocrystals obtained after a reaction temperature of 30 min and 3 h in air; (**b**) Schematic of crystal structure during phasic transformation of tin oxide to tin dioxide; (**c**) Bright-field TEM image and HRTEM (inset) of SnO nanosheets; (**d**) SAED image of SnO nanosheets; (**e**) Bright-field TEM image and HRTEM (inset) of SnO_2_ nanoparticles; (**f**) SAED image of SnO_2_ nanoparticles.

To completely understand the formation of SnO_2_ nanoparticles in air, we have observed our results after 30 min and 3 h. We obtained square nanosheets with average dimensions of 6 μm × 6 μm × 0.1 μm after 30 min in air with the HRTEM image revealing an interplanar distance of 0.272 nm corresponding to the (110) plane of SnO (Figure 1c). The SAED pattern reveals a single crystal structure and the diffraction spots can be indexed to the planes of SnO. However after 3 h in air, the SnO nanosheets disintegrate to form SnO_2_ nanoparticles with an average diameter of about 5–7 nm (Figure 1e). The HRTEM image of the nanoparticles shows a crystal structure with an interplanar distance of 0.323 corresponding to the (110) plane of SnO_2_. The SAED data reveals a polycrystalline structure and the rings can be indexed to crystal planes of pure SnO_2_ (Figure 1f). The results observed under TEM agree with the PXRD data (Figure 1a) and confirm the transformation of tetragonal SnO nanosheets (a = b = 0.3802 nm, c = 0.4836 nm, JCPDS card No. 06-0395) to tetragonal-rutile SnO_2_ nanoparticles (a = b = 0.4737 nm, c = 0.3186 nm, JCPDS card No. 41-1445) where each Sn atom is surrounded by a distorted octahedron of oxygen atoms. The sharp peaks indicate that the SnO nanosheets are highly crystalline and the particle size is large. In addition, the diffraction intensities of the (001) and (002) planes have a higher relative intensity ratio than the standard powder diffraction file which implies that the synthesized powder has a preferred crystal orientation. The average crystallite size calculated by the Scherrer’s peak equation for SnO_2_ nanoparticles was found to be 7 nm, thus further confirming our TEM results.

**Figure 2:**
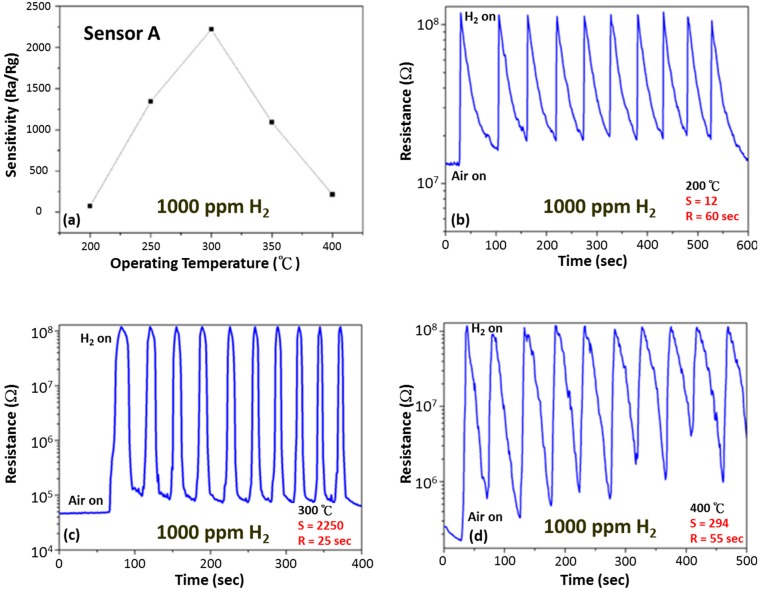
(**a**) Gas sensitivity as a function of operating temperature for pure SnO_2_ thick film sensor. Change in resistance of pure SnO_2_ gas sensors on exposure to 1000 ppm of H_2_ for 10 cycles at an operating temperature of (**b**) 200 °C, (**c**) 300 °C and (**d**) 400 °C.

In our previous report [26] we studied the transformation of the tin oleate precursor under nitrogen (N_2_) atmosphere and found that the SnO changes morphology from tetrahedral (30 min) to decahedral (3 h) but does not transform structurally into SnO_2._ On the basis of the experimental results, we can propose a mechanism for the formation of SnO_2_ nanoparticles. As the TOA was added into the tin oleate complex solution and heated to 340 °C, the tin oleate decomposed into SnO monomers. The coordinating solvent TOA works as an activator to trigger the decomposition reaction as well as a surfactant to control the shape of the SnO particles. The SnO monomers are like building blocks which assemble into larger SnO nanosheets to minimize the surface energy. When TOA is absorbed isotropically on the surface in the presence of N_2_, it is unstable in air and selectively absorbs on the (001) plane and thus the two reaction conditions result in different morphologies of SnO. Heating in air for 3 h causes the SnO nanosheets to transform into the more energetically stable SnO_2_ phase. The synthesized pure SnO_2_ nanoparticles were made into a thick film sensor (Sensor A) as described in the experimental section and the sensitivity and response time measurements at different operating temperatures can be shown in Figure 2.

Sensor A shows a highest sensitivity of 2250 for 1000 ppm H_2_ at an operating temperature of 300 °C with a sensor response time of about 25 s and a good reversibility over several cycles of H_2_ exposure. However, the sensor response is strongly temperature dependent, showing very low sensitivities at temperatures below 200 °C and above 400 °C. The oxygen present in air is absorbed on the SnO_2_ nanoparticle surface by different mechanisms depending on the operating temperature, from physisorption (molecular form) to chemisorption (dissociative form) as the temperature increases [27]. This explains why low sensitivity is observed below 200 °C where the physisorption of oxygen molecules dominates. As the temperature, increases to 300 °C, chemisorption of oxygen results in more free electrons being extracted by the adsorbed oxygen, resulting in an increase in initial resistance and thus an improved overall sensor response. Further increase in temperature up to 400 °C leads to a reduction of the trapped metastable electrons from the conduction band due to desorption of oxygen species and hydroxyl groups, resulting in a fall in the initial resistance and hence reducing the overall sensor response.

The electrical properties of nanocrystalline SnO_2_ strongly depend on the nanoparticle size and the surface state produced by gas adsorption resulting in space charge and energy band modulation. This adsorption of oxygen species creates an electron depletion layer which results in decrease in carrier concentration and an increase in the surface potential barrier [28,29,30,31]. When the sensor is exposed to a reducing gas like H_2_, the surface oxygen is consumed and this donates electrons back to the surface of the semiconductor oxide, decreasing the Schottky barrier height and thus causing a fall in resistance of the sensor film. The advantage of having the sensor layer made of very small nanoparticles on the order of the thickness of the charge depletion layer as used in this study also improves sensor response. To achieve maximum gas response the grain size of the sensing nanomaterial must be smaller than twice the size of the depletion region (D~2 L, where D is the particle diameter and L is the Debye length). A faster diffusion rate of the hydrogen and oxygen species can also be achieved if the sensor film has a highly porous microstructure, resulting in a faster response and recovery time. The sensing mechanism of metal oxide semiconductors is based on the change in resistivity due to a change in carrier concentration and mobility on exposure to the test gas. The neck control model can be used to explain this mechanism by indicating that the carrier concentration is controlled by the depletion layer and the mobility is limited by the width of the conduction channel at the grain neck [32,33,34]. However because of the absence of surface loaded catalysts which initiate the spill-over mechanism, pure SnO_2_ sensors are unsuitable for operation at temperatures lower than 200 °C with extremely low sensitivities and very long response times observed at room temperature.

### 3.2. Pt-Loaded SnO_2_ Nanoparticle Based Sensor

**Figure 3:**
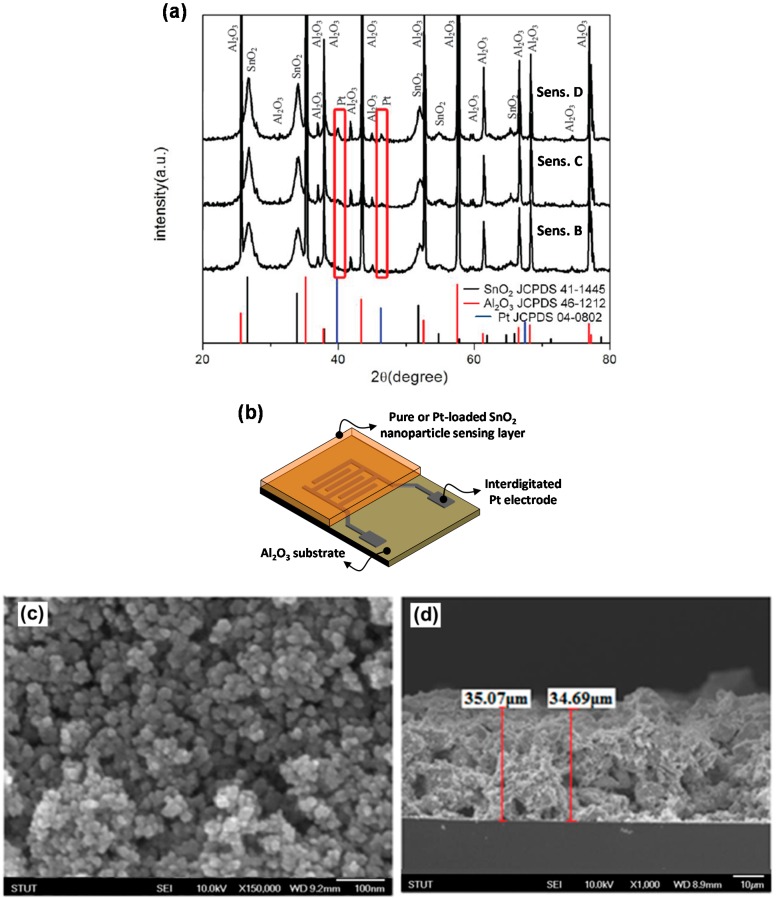
(**a**) XRD result of sensing film loaded with different concentrations of Pt; (**b**) Schematic illustration of thick film sensor utilizing interdigitated Pt electrodes. SEM image showing (**c**) top view and (**d**) cross sectional morphology of sensing film.

As mentioned in the experimental section, we loaded the SnO_2_ nanoparticles with three different concentrations of Pt (Sensor B, C and D). The Pt-loaded SnO_2_ thick film sensor was fabricated using the same screen printing and annealing steps utilized for the pure SnO_2_ thick film sensor (Sensor A). Sensors were characterized using XRD while the top and cross sectional morphology was observed under an SEM as shown in Figure 3. The XRD results show peaks corresponding to the (111) and (200) planes of Pt and the intensity of the peaks increases as the Pt-loading concentration increases. The SEM images show a highly porous interconnected structure with an average sensor film thickness of about 35 μm.

**Figure 4:**
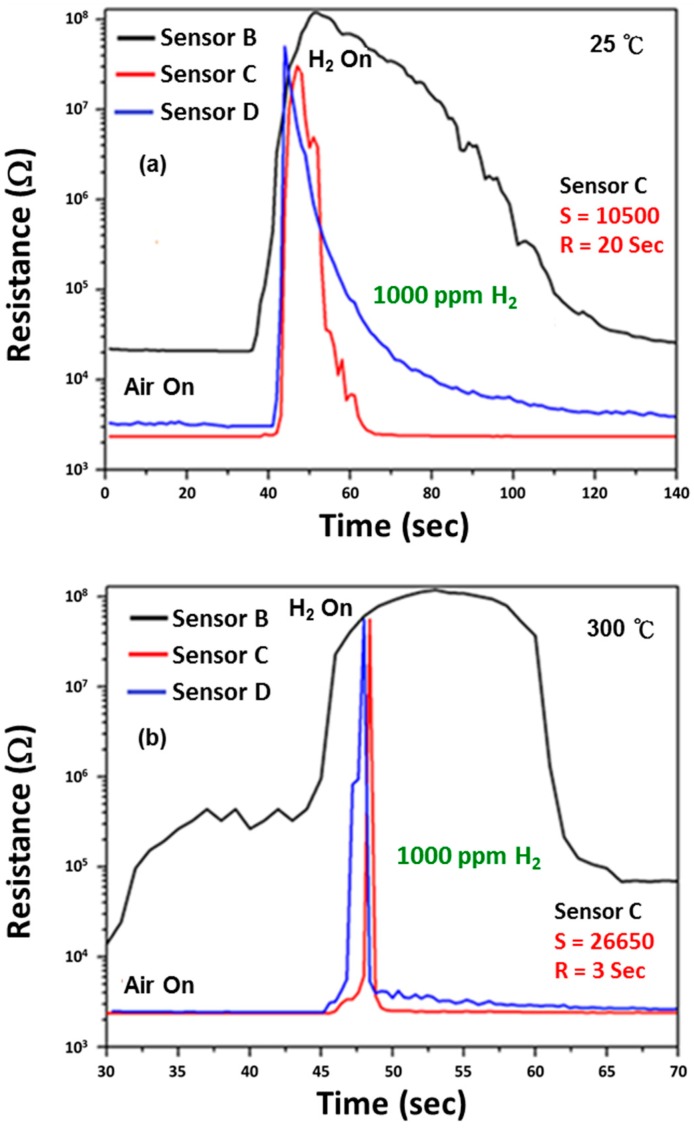
Change in resistance of SnO_2_ gas sensors with different Pt loading concentrations upon exposure to 1000 ppm of H_2_ gas at an operating temperature of (**a**) 25 °C and (**b**) 300 °C.

Lower operating temperatures are crucial if the sensors are to be energy efficient, stable and safe. Sensors fabricated using Pt-loaded SnO_2_ nanoparticles show a highest sensitivity of 26,650 (Sensor C) with a response time of 3 s at 300 °C (Figure 4b). While the sensor sensitivity after Pt-loading was highly enhanced at higher operating temperatures, it also shows a high sensor response of 10,500 at room temperature with a response time of about 20 s which is more than four times higher than the sensor sensitivity of 2250 obtained using pure SnO_2_ at 300 °C. This is because the sensing performance at lower working temperatures strongly depends on the deposited Pt-nanoparticles rather than the oxide support. It has been observed that Pt nanoparticles with smaller sizes, narrow size distribution and more uniform and isolated deposition on the oxide surface results in improved sensing results [35]. Moreover, it has been observed that sensor sensitivity and response time decreases for Sensor B and Sensor D as compared to Sensor C. This is because it is only at a certain loading concentration when the Pt nanoparticles are dispersed homogeneously in the SnO_2_ matrix and can effectively catalyze the reaction between oxygen and hydrogen on the SnO_2_ surface via the spill-over effect. The term spill-over refers to the process during which the metal catalyst dissociates the adsorbed gas molecules which can then spill-over onto the surface of the semiconductor. The H_2_ gas is first absorbed on the surface of the Pt nanoparticles and then migrates to the surface of the SnO_2_ primary nanoparticle where it reacts with the absorbed oxygen species.

The role of Pt-loaded surface catalysis in oxidation of H_2_ causes electrons to be injected into the primary SnO_2_ nanoparticle and thus explains the decrease of the depletion layer width and expansion of the conduction channel. For loading concentrations of Pt higher than the optimum limit (as in the case of Sensor D), the nucleation type will change from homogeneous to heterogeneous with agglomeration of Pt nanoparticles. In this case, the spill-over effect by Pt becomes less effective as less H_2_ can be dissociated and spilled over onto the SnO_2_ surface. Furthermore, agglomeration reduces the total surface area for gas sensing interaction, thus leading to lower sensitivity and higher response times Thus it is vital that the Pt catalyst is dispersed uniformly on the SnO_2_ surface to ensure effective migration of absorbed H_2_ species to the interparticle contact. It is also important that the nanoparticulate ink after screen printing must be annealed at sufficiently high temperatures to provide an interconnected structure which reduces the height of the energy barrier between neighboring SnO_2_ grains, thus improving sensor sensitivity. The annealing atmosphere and temperature also affects the chemical state of the loaded Pt [36] and the dispersion nature of Pt at the SnO_2_ surface. It was observed that Pt (II) shows much higher activity than Pt (IV) in catalyzing the dissociation and adsorption reaction on the SnO_2_ surface. In summary, the spill-over model helps to explain the improved sensitivity and response time observed when loaded with Pt. At higher working temperatures some of the oxygen species will be adsorbed on the secondary Pt nanoparticles which will reduce the probability of H_2_ dissociation. This negative temperature dependence of Pt-doped SnO_2_ sensors makes them much more suitable for room temperature operation as compared to sensors fabricated using pure SnO_2_.

## 4. Conclusions

In this study we have successfully synthesized a Pt-loaded SnO_2_ nanoparticle sensor which exhibits high sensitivity to H_2_ gas and fast response time at room temperature. The highly porous interconnected thick film structure enhances the surface exposure area and hence the sensor sensitivity. The mechanism for H_2_ sensing was analyzed based on the variation of conduction channels and can be explained by the neck control model for pure SnO_2_ and the spill-over effect for Pt-loaded SnO_2_ nanoparticles. While room temperature sensitivity can be achieved, further batch tests need to be carried out to ensure the stability and reproducibility of these sensors over longer time periods. These sensors can be used for real time monitoring of H_2_ gas at ambient temperatures with high stability and low power consumption.
